# Moringa oleifera leaf extract promotes the healing of critical sized bone defects in the mandibles of rabbits

**DOI:** 10.1038/s41405-024-00201-y

**Published:** 2024-03-14

**Authors:** Nouran A. Elsadek, Maha A. Aboukhadr, Fatma R. Kamel, Hossam M. Mostafa, Gillan I. El-Kimary

**Affiliations:** 1 Department of Oral Medicine, Periodontology, Oral Diagnosis and Oral Radiology, Faculty of Dentistry, Alexandria University, Alexandria, Egypt; 2 Department of Oral Biology, Faculty of Dentistry, Alexandria University, Alexandria, Egypt

**Keywords:** Periodontitis, Dental biomaterials

## Abstract

**Objective:**

The search for an osteopromotive material that enhances the efficacy of alloplasts in reconstructive surgeries has been going on for years. This study aimed to histologically and histomorphometrically evaluate the efficiency of Moringa oleifera leaf extract as an osteopromotive biomaterial.

**Design:**

The study is a prospective randomized controlled animal study. 24 adult male New Zealand rabbits were equally allocated into test and control groups. Critical-sized bone defects were created in the edentulous areas of the mandibles of rabbits. The defects of the control group were filled with Beta-tricalcium Phosphate, while the defects of the test group were filled with Beta-tricalcium Phosphate combined with Moringa oleifera leaf extract. The results were evaluated histologically and histomorphometrically.

**Results:**

Histological and histomorphometric analysis showed a significant increase in the surface area of bone and the number of osteoblasts in test groups compared to those in the control groups.

**Conclusion:**

Moringa oleifera leaf extract has a positive effect on bone regeneration in critical-sized bone defects.

## Introduction

Periodontal disease includes a broad spectrum of symptoms and manifestations, involving hard and soft tissues, that oftentimes acts as a cascade eventually leading to bone loss [1]. The mandible is a bone that is actively integral in speech and mastication and passively maintains the shape of the face [2]. Large mandibular defects may result from trauma, infection, tumors managed with resection or periodontitis [3]. Large defects that wouldn’t heal on their own present a challenge to clinicians all over the world.

A critical-sized defect is the smallest defect that won’t spontaneously heal on its own through the duration of the experiment nor through the animal’s lifetime [4].

As bone defects greatly affect mastication, and occasionally esthetics, slow regeneration could impose a therapeutic problem. One of the methods to overcome this problem is combining bone graft with osteopromotive components that enhance new bone formation such as herbal extracts [5]

Moringa oleifera (MO) leaves contain various flavonoids that can induce osteogenic differentiation of mesenchymal stem cells derived from bone marrow [6]. Flavonoids protect cells from oxidative stress by scavenging free radicals [7]. Moringa oleifera leaves contain antibacterial phytochemicals as Pterygospermin, Benzyl Isothiocyanate and Benzyl Glucosinolate [8]

Moringa oleifera has been widely tested as an oral care product in humans. One study was conducted to assess the effect of Moringa oleifera leaf extract in patients suffering from Early Childhood Caries. It was found that gargling with Moringa oleifera leaf extract effectively diminishes plaque formation [9]

A study conducted by Patel et al. [10] has proved that flavonoids and other components in Moringa oleifera have positive osteoblastic stimulating potential. A study conducted by Djais et al. [11] evaluating the effect of combining Moringa oleifera with Demineralized Freeze Dried Dentin Matrix (DFDDM) in socket preservation suggested that the combination can effectively generate fibroblast and osteoblast expressions. Moringa oleifera effectively regulates the p38α/MAPK14-OPG/RANKL pathway [12]. It does not only alter the expression of inflammatory cytokines, but Moringa oleifera leaf extract also substantially reduces alveolar bone resorption [12]

Pure β-TCP is a popular choice for monitoring healing due to its availability and ease of handling [13]. A systematic review and meta-analysis published in 2021 that included 5 studies showed that β-TCP used to manage infrabony defects resulted in positive outcomes regarding bone fill and gain of clinical attachment. However, when used with growth factors, β-TCP showed superior results to those shown when used alone [14]

Animal models allow researchers to histologically assess the processes of bone remodeling and regeneration on a cellular level to understand the course of healing and the effect of regenerative materials. The results of this study could contribute to a better quality of life for thousands of people using a biocompatible, cost-effective and widely available material that could enhance the healing process of bone.

In our study, the effect of combining β-TCP with Moringa oleifera leaves extract on healing of critical sized bone defects in rabbits was assessed.

## Materials and methods

### Study design and sample size

This study is a prospective randomized controlled animal study. The authors followed ARIVE guidelines 2.0.

**Fig. 1:**
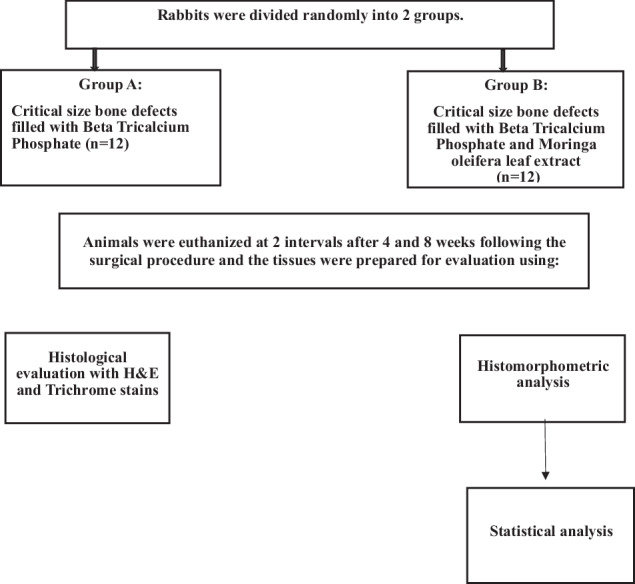
Flowchart of the study design showing the random allocation of the rabbits into test and control groups, the follow-up periods, and the methods of evaluation. Rabbits were randomly divided into a control group and an experimental group, in which each group included 12 New Zealand rabbits. Animals were then euthanized and mandibles were prepared for histological evaluation and histomorphometric evaluation for statistical analysis.

Sample size was estimated assuming alpha error = 5% and study power = 80%. Djais et al. [11] reported mean ± SD osteoblasts number after 21 days = 90.08 ± 8.07 when moringa was used, and 113.3 ± 12.50 when moringa was combined with freeze dried dentin matrix graft Fig. 1. Based on comparison of means, sample size was calculated to be 5 per group, increased to 6 to make up for laboratory processing errors. The total sample size = number of groups × number per group × number of timepoints = 2 × 6 × 2 = 24.

### Ethical statement

The study was performed after gaining the approval of the Research Ethics Committee, Faculty of Dentistry, Alexandria University.

All animals’ procedures followed the National Research Council Guidelines for the care and use of laboratory animals [15].

### Randomization and experimental procedure

Random allocation was done, by using a computer-generated random sequence of numbers to assign treatment status to decrease the risk of confounding. Randomization was conducted by giving each defect used in this study a number from 1 to 24 and using computer assisted software; 12 rabbits were randomly allocated to each of the 2 groups. Half of each group were euthanized after 4 weeks, and the other half after 8 weeks.

### Preparation of the aqueous extract of Moringa oleifera leaves

Fresh Moringa oleifera leaves were left to dry for 7 days, then infused in distilled water (100 glL). The mixture was then boiled for 20 min then filtered out [16].

24 adult male New Zealand rabbits of 6–7 months of age, weighing 2.5–3.5 kgs were supplied from the national institute of research.

General anesthesia was induced by an intramuscular injection of a combination of 25 mg/kg weight ketamine and 5 mg/kg body weight xylazine.

**Fig. 2:**
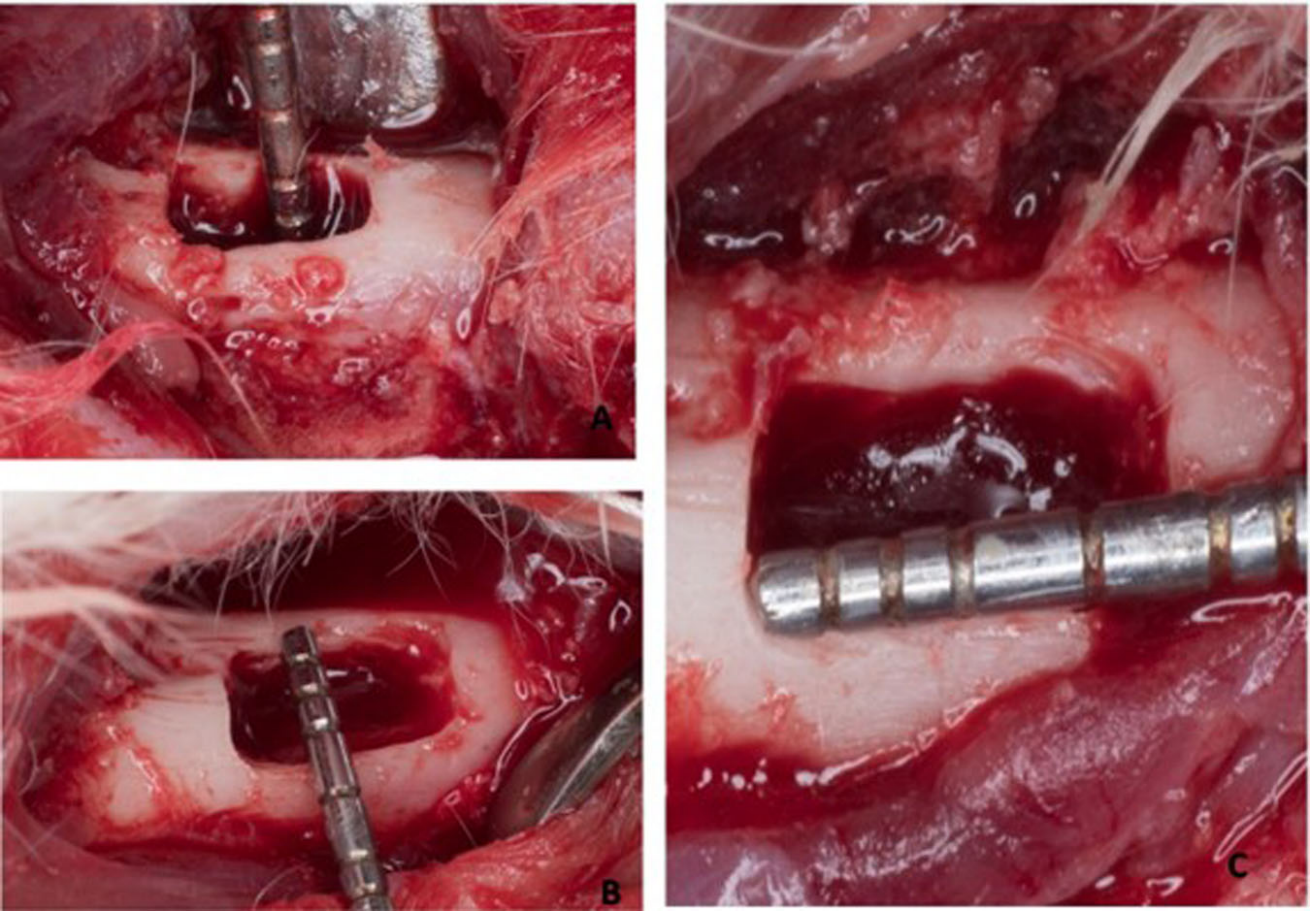
The dimensions of the surgically induced standardized critical sized defects performed in 24 defects. **A** 3 mm apico-coronal dimension. **B** 6 mm mesiodistal dimension. **C** 4 mm buccolingual dimension.

The edentulous alveolar ridge on the right side of the body of the mandible between the incisor and the first posterior tooth of each animal was selected for the surgical site. A full mucoperiosteal flap was raised intraorally. Osseous defects measuring 6 mm (mesiodistal) ×4 mm (buccolingual) ×3 mm (apicocoronally) were prepared in the edentulous diastema [17, 18] (Fig. 2)_._

The osseous defects were washed out with sterile saline to remove any bone particles that could possibly initiate osteoinduction [19]. Defects were filled with β-TCP in the control group, and filled with a combination of β-TCP and Moringa oleifera leaves extract in the test group.

Flaps were then repositioned and sutured with 3-0 black silk suture. To control postoperative swelling and pain, a subcutaneous injection of 4 mg/kg Carprofen injection was administered twice daily for four consecutive days.

Animals were euthanized using an overdose intravenous injection of pentobarbital 120 mg/kg (Streuli Pharma AG, Uznach, Switzerland) [20].Disposal of animals was done through incineration.

### Blinding

Concealment of group allocation from the researcher performing the histological examination ensured a single-blind study and made the results of the study less likely to be biased.

### Outcome measures

#### Histological assessment

Bony specimens were fixed with 10% freshly prepared formalin for 24 h then decalcified using 10% formic acid for 7 days. Embedding was done using paraffin wax; bone blocks were sectioned by microtome for serial sections of 4 μm. Sections were placed on slides and stained using hematoxylin and eosin (H&E) stain, and Trichrome stain. The morphological characters of the newly formed bone were evaluated.

#### Histomorphometric assessment

10 images at ×100 magnification and 10 images at ×200 magnification were taken for every specimen under light microscope.

The following variables were assessed using Image analysis software J 1.46r [21] software:

1. The percentage of the surface area of newly formed bone compared to the total surface area of the surgically induced critical sized defect.
2. Osteoblastic count: the number of osteoblasts in each photomicrograph was counted.

### Statistical methods

Descriptive statistics were calculated as means, standard deviation (SD) and range. Normality was checked using descriptive statistics, plots (Q-Q plots and histograms), and Shapiro Wilk normality test. All variables showed normal distribution, so parametric tests were used. For normally distributed data, comparison between two independent population were done using independent t-test, while the comparison between the same group at a different period of time was done using paired t-test.

The significance level was set at *p* value < 0.05. Data were analyzed using IBM SPSS software package (Version 24.0).

## Results

### Histological results

#### Four-week follow up results

**Fig. 3:**
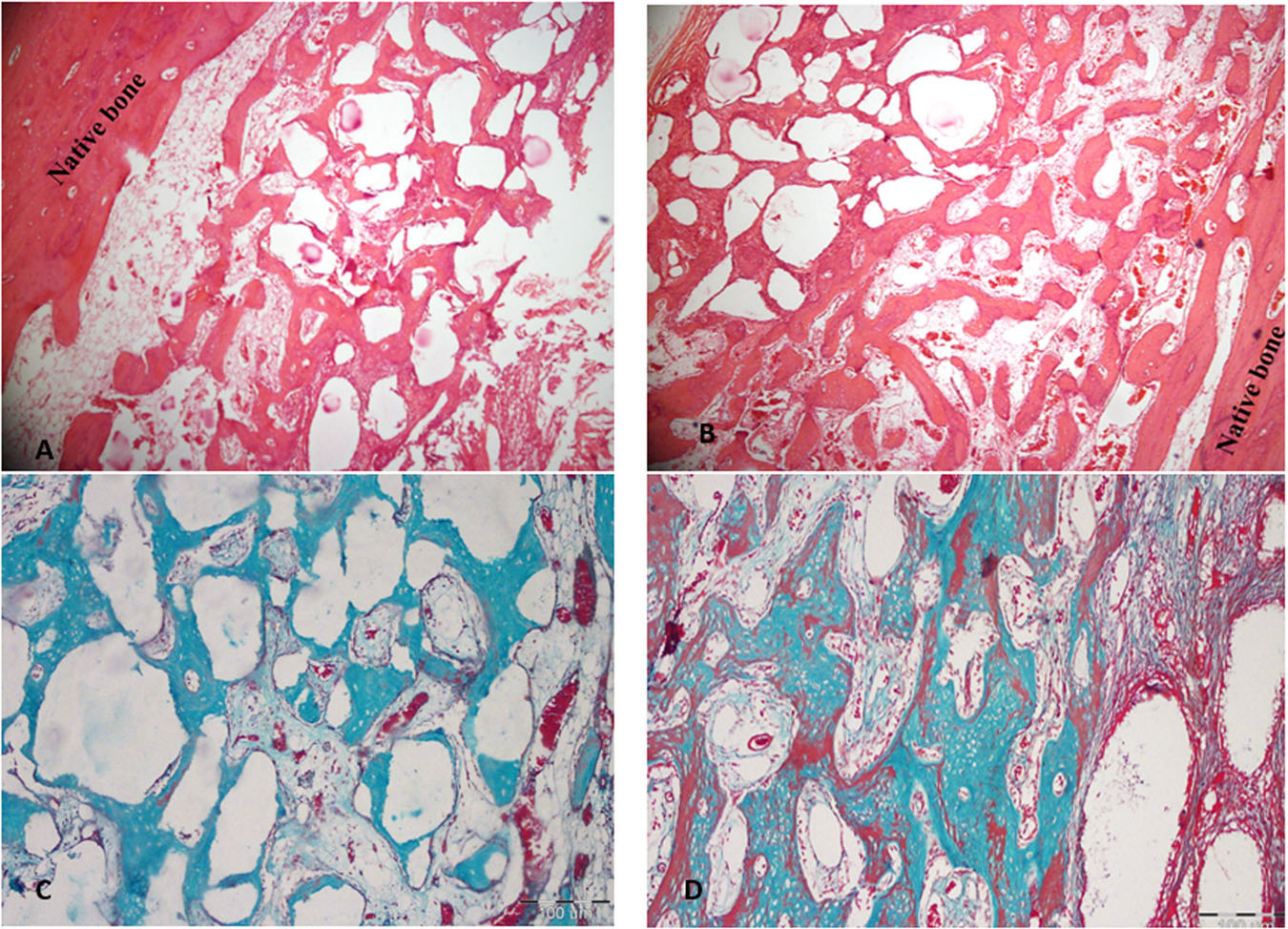
Histologic differences between experimental and control groups 4 weeks post-operatively. **A** Control group showing numerous blood vessels containing red corpuscles of red blood cells are found in the marrow spaces surrounding the newly formed bone trabeculae. (H&E; original magnification ×100). **B** Test group shows a huge amount of blood vessels with marrow spaces surrounding the trabeculae of the newly formed bone (H&E; original magnification ×100). **C** Control group light micrograph showing mature bone within defect with wide marrow spaces of moderate vascularity (*Masson’s* trichrome; original magnification ×100). **D** Test group light micrograph showing newly formed bone lined by active osteoblast. Fibrous mapping of newly formed trabeculae is also noticed. (*Masson’s* trichrome; original magnification ×100).

Four weeks postoperatively, the surgical defects of the control group were filled with bone trabeculae of moderate thickness and amount (Fig. 3). Numerous blood vessels containing red corpuscles of red blood cells were found in the marrow spaces surrounding the newly formed bone trabeculae.

In the test group, the entire defect was filled with bone trabeculae of greater amount and thickness than the control group. Primary osteons were clearly visible in various areas within the newly formed bone lined by a dense layer of voluminous osteoblasts and surrounded by highly vascular marrow spaces. Also, reversal lines were clearly seen within the newly formed bone trabeculae.

Trichrome stained sections demonstrating the histological outcomes at 4 weeks time point show interconnected trabeculae formation of different sizes of immature bone separated by considerable amounts of Beta Tricalcium Phosphate for the control group. Greater amount of bone is formed in the test group than in the control group. The formed trabeculae are thicker and more inter-communicated. Considerable blood supply is also seen between intervening connective tissue.

#### Eight-week follow up results

After eight weeks, the surgical defects of the control group are filled with bone trabeculae of various thickness and maturity. The formed bone enclosed particles of bone grafts and is surrounded by dense fibrous marrow spaces.

**Fig. 4:**
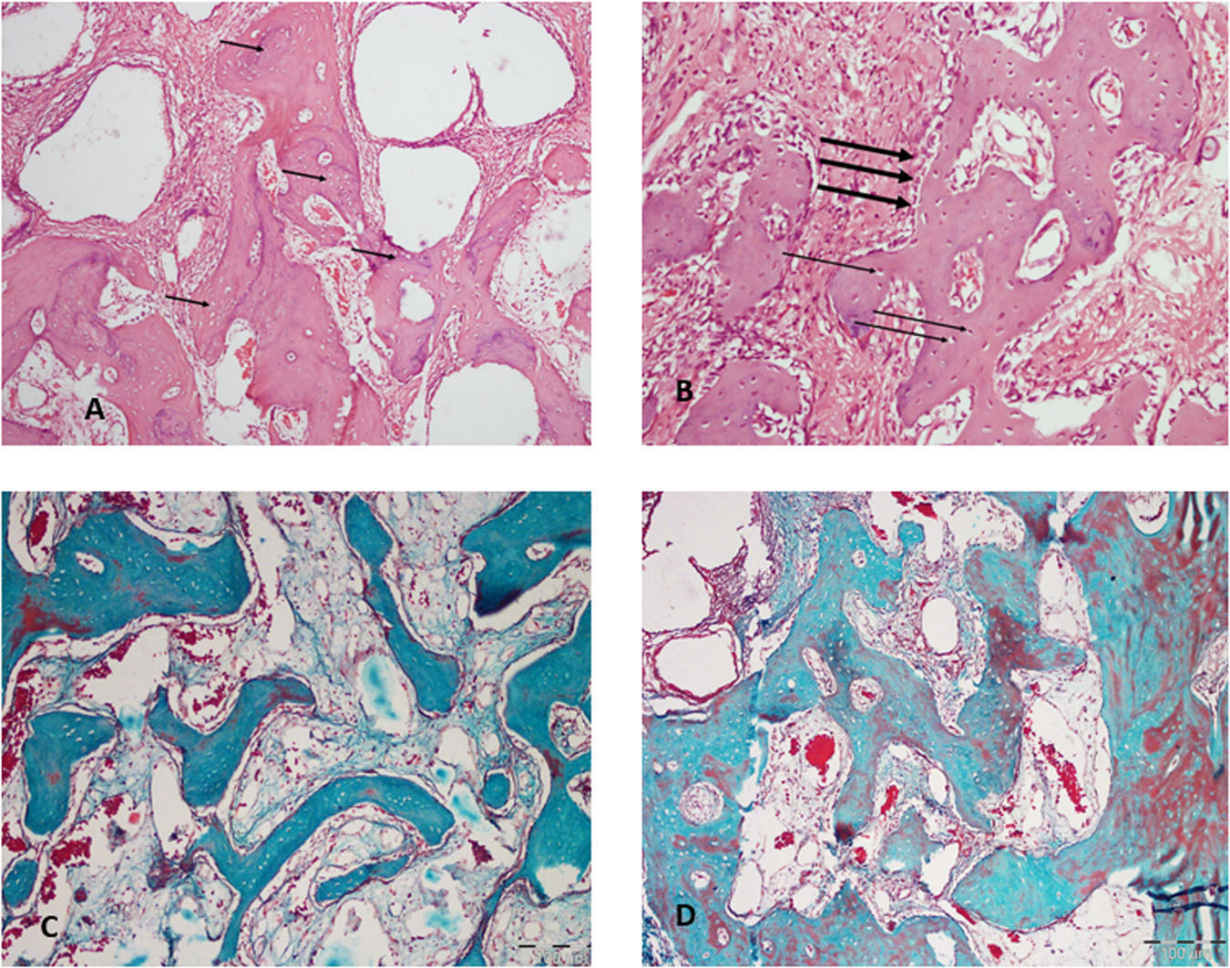
Histological differences between test and control groups 8 weeks postoperatively. **A** Control group shows the intermingle between graft particles (arrows) and the formed mature bone. (H&E; original magnification ×100). **B** Test group shows the newly formed bone that is lined by active plump osteoblasts (thick arrow) and contains numerous osteocytes (thin arrows). (H&E; original magnification ×100). **C** Light micrograph of the control group showing bone trabeculae within defect with marrow spaces of moderate vascularity (*Masson’s* trichrome; original magnification ×100). **D** Light micrograph of the test group showing mature bone within the defect, the marrow spaced show evident fibrous tissue and moderate vascularity. (*Masson’s* trichrome; original magnification ×100).

The test group shows inter-communicated dense bone trabeculae filling the entire surgical defect. Remnants of bone graft particles are seen within bone trabeculae lined by numerous plump osteoblasts and enclose numerous osteocytes (Fig. 4A, B).

Figure. 4C, D shows trichrome stained sections demonstrating the histological outcomes at 8 week-time point. The control group shows thin bone trabeculae that are not interconnected and separated by broader surface area of intervening connective tissue. The test group resulted in greater amounts of intercommunicated bone trabeculae that are thicker than those presented in the control groups.

### Histomorphometric analysis

The percentage of newly formed bone in relation to the total surface area of the defect was calculated in all groups. The mean percentage of newly formed bone is illustrated in Table 1. After four weeks, the mean percentage of surface area of bone formed was 19.42% in the control group and 31.05% in the test group, yielding a significant difference between both groups with a 0.001 *p*-value. Likewise, the results studied at 8-week time point resulted in a significant difference with a 0.028 *p*-value between the test and control groups as the mean percentage of surface area of bone was 33.78% for the control group and 41.12% for the test group.

**Table 1:** Histomorphometric results of the percentage of surface area of bone in relation to the total surface area of the defect.

Percentage of bone formed in relation to the total surface area of the defect.	Control group	Test group	Unpaired t-test	*P* value
After 1 month				
Range	16.77–23.36	27.72–38.34		
Mean	19.42	31.05	11.03	0.001^a^
S.D.	2.70	3.72		
95.0% C.I.	17.1–21.6	27.9–34.1		
After 2 months				
Range	25.07–39.92	33.34–44.99		
Mean	33.78	41.12	2.01	0.028^a^
S.D.	4.50	3.93		
95.0% C.I	30.0–37.5	37.8–44.4		
Paired t-test	12.65	5.21		
*P* value	0.001^a^	0.002^a^		

Unpaired t-test was used to compare between the two studied groups at the same time.

Paired t-test was used to compare between the interval times in the same group.

*P* was significant if ≤0.05.

^a^Significant difference.

**Fig. 5:**
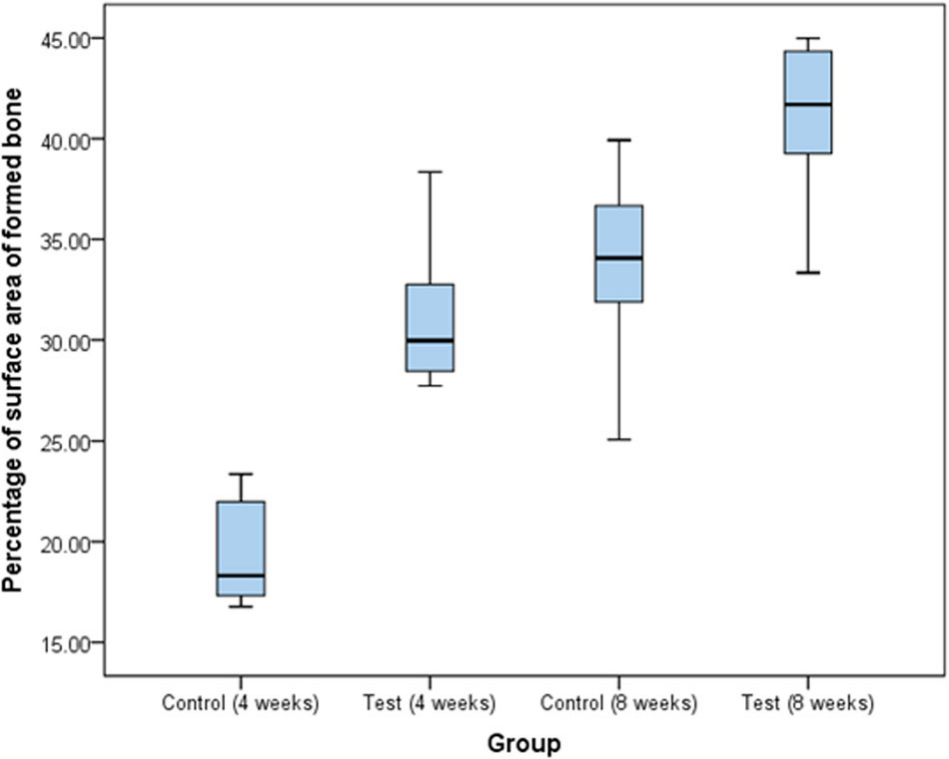
Graph shows the significant difference in the percentage of the surface area of bone in comparison to the total surface area of the defect after 4 and 8 weeks in both the test and control groups with 95% confidence interval. After 1 month, the range of percentage of surface area of bone in comparison to the total surface area of the defect in the control group ranges between 16.77% and 23.36% with a mean of 19.42%, while in the test group the percentage ranged from 27.72% to 38.34%. After 2 months, the range of percentage of surface area of bone in comparison to the total surface area is from 25.07% to 39.92% with a mean of 33.78% for the control group while for the test group the percentage ranged from 33.34% to 44.99% with a mean of 41.12%.

The graph in Fig. 5 summarizes the results of our study by demonstrating the mean percentage of surface area of bone formed in relation to the total surface area of the defect.

The osteoblastic count was studied by quantitative assessment of the histologic photomicrographs of all study groups using image J software, as represented in Table 2. After 4 weeks, the mean number of osteoblasts per photomicrograph was 77 for the control group and 101 for the test group, yielding a significant difference between both groups with a 0.001 *p*-value. Likewise, after 8 weeks, the mean number of osteoblasts for the control group was 61.71 and 70.43 for the test group, yielding a significant difference between both groups with a 0.003 *p*-value.

**Table 2:** comparison between osteoblast count per photomicrograph after 4 and 8 weeks in test and control groups.

Osteoblast count per photomicrograph	Control group	Test group	Unpaired t-test	*P* value
After 4 weeks				
Range	69–81	97–108		
Mean	77.00	101.86	7.98	0.001^a^
S.D.	4.04	4.06		
95.0% C.I.	73.3–80.7	98.1–105.6		
After 8 weeks				
Range	56–68	67–75		
Mean	61.71	70.43	4.21	0.003^a^
S.D.	4.46	2.82		
95.0% C.I.	57.6–65.8	67.8–73.03		
Paired t-test	6.21	8.21		
*P* value	0.002^a^	0.001^a^		

Unpaired t-test was used to compare between the two studied groups at the same time.

Paired t-test was used to compare between the interval times in the same group.

*P* was significant if ≤0.05.

^a^Significant difference.

**Fig. 6:**
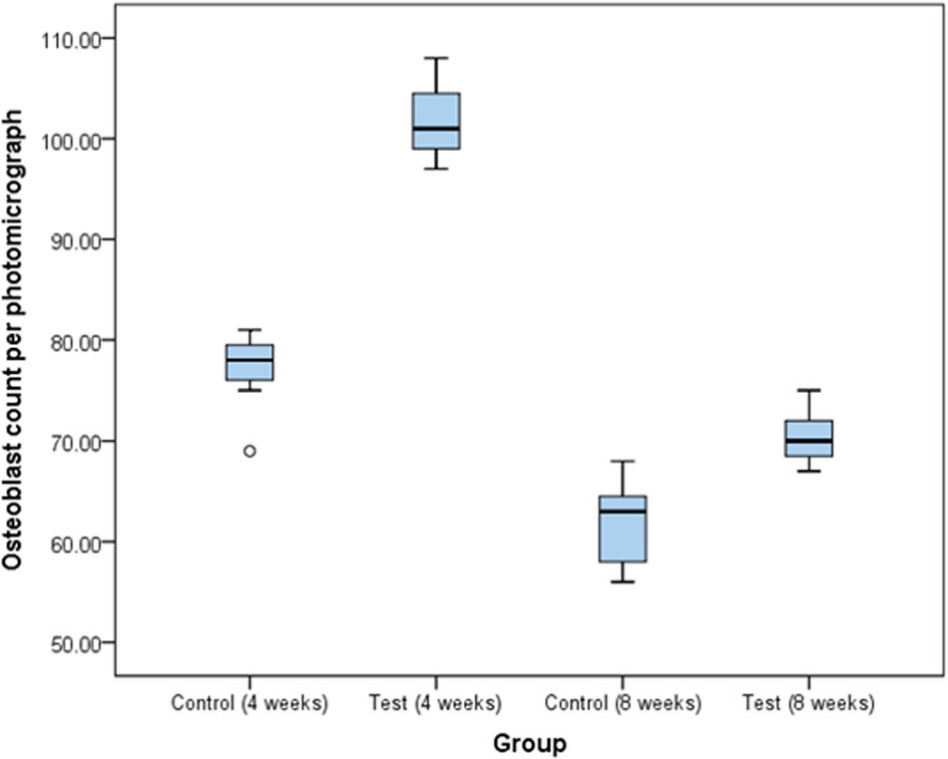
Comparison between Osteoblast count per photomicrograph in the study and control groups after 4 and 8 weeks with 95% confidence interval. The figure shows that the range of number of osteoblasts per photomicrograph for the control group after 1-month ranges from 69 to 81 with a mean of 77 osteoblasts while the number of osteoblasts per photomicrograph for the test group after 1 month ranged from 97 to 108 with a mean of 101.86. After 2 months, the number of osteoblasts per photomicrograph for the control group ranged from 56 to 68 with a mean of 61.71 while for the test group it ranged from 67 to 75 with a mean of 70.43 osteoblasts per photomicrograph.

The graph in Fig. 6 demonstrates the mean number of osteoblasts counted per photomicrograph for all study groups.

## Discussion

In the current study, we investigated the effect of Moringa oleifera, a plant-based traditional medicine, in the management of critical-sized bone defects in the mandibles of rabbits.

We chose rabbits as they are considered the most convenient small animal that allows histological examination of bone regeneration for multiple reasons. The first reason is that the mandibles of rabbits are larger than those of rats, making the surgical procedure technically more feasible and reproducible [22]. More importantly, rabbits manifest multicellular unit remodeling highly similar to the remodeling that occurs in humans [22].

Most studies assess periodontal regenerative therapy through clinical and radiographic evaluation. However, we chose to conduct a histological and histomorphometric experimental study. This is the first study to assess the effect of Moringa oleifera leaf extract in combination with Beta-tricalcium Phosphate in critical-sized intra-oral bone defects. The monocortical critical-sized defects performed in this study allowed us to examine the direct effect of Moringa oleifera leaf extract on bone filling in a simple and standardized manner [23].

Histomorphometry is the quantitative assessment of histological samples. It has long been proven to be a powerfully valid tool widely used to assess bone regeneration [24].

Our study revealed that Moringa oleifera leaf extract substantially increased the percentage of the surface area of bone in relation to the total surface area of the surgically induced critical-sized defect. The results of our study are consistent with the results of a study held in 2019, in which the expression of osteocalcin and Transforming Growth Factor Beta1 (TGF-β1) was studied. The participants of this study were divided into groups; all of which underwent extraction of the left mandibular incisor. The mean values for the expression of osteocalcin and TGF-β1 were higher in the groups treated with Moringa oleifera leaf extract, Demineralized Freeze Dried Bovine Bone Xenografts (DFDBBX) and Polyethylene glycol (PEG) than for the groups treated with DFDBBX and PEG alone [25] One reason for these results may be attributed to is the myriad amount of saponins and flavonoids found in Moringa oleifera.

In 2008, an in vitro study assessed the activity of Moringa oleifera leaf extract as a free radical scavenger and concluded that it has a potential therapeutic antioxidant effect [26]. Moringa oleifera has also been showing anti-inflammatory effects by reducing the production of Interleukin-6 (IL-6), a pro-inflammatory cytokine induced by Porphyromonas gingivalis [27].

We also found that the addition of Moringa oleifera leaf extract to alloplastic bone graft significantly increased the number of osteoblasts in the defect, especially in the proliferative stage. These results come in accordance with a study held by Jeong et al that found that saponins have an in vitro osteogenic action that affects osteoblastic proliferation and differentiation [28]. Flavonoids in Moringa oleifera play an important role in bone repair, but kaempferol flavonoids have been proven to be of special importance. Kaempferol activates estrogen receptors inducing osteoblasts differentiation [29].

Moringa leaves possess a considerably high amount of tannins ranging from 13.2 to 20.6 g tannin/kg of dried leaves [30]. These tannins can inhibit osteoclast differentiation, which in turn facilitates new bone formation [31].

In 2020, research was conducted to investigate the effect of Moringa oleifera leaf extract on orthodontic tooth movement after its administration in tension areas. It was concluded that Moringa oleifera leaf extract substantially increased the number of osteoblasts and decreased the number of osteoclasts in tension areas where it was administered [32].

Another study was held by Khan et al. [33] in 2022 in which the effect of Moringa oleifera leaf extract on osteoblast cell differentiation was examined. This study used Alkaline phosphatase analysis, which concluded that Moringa oleifera leaf extract increases osteoblast cell differentiation and enhances the expression of both Runt-related transcription factor 2 (RUNX2) and Bone morphogenetic protein 2 (BMP2) genes when used in a concentration of 25 or 50 μg /mL. Marupanthorn et al. [34] conducted a study that demonstrated that Moringa oleifera leaf extract potentiates osteogenic differentiation of bone marrow-derived stem cells in pigs.

Among the limitations of our study is the lack of longer follow-up periods, and that the study was conducted on a small sized animal. In the future, it is recommended to test the efficacy of Moringa oleifera leaf extract on bone healing on larger sample sizes of bigger sized animals and in randomized controlled clinical trials. The use of the critical sized defect model in this experiment ensures the reliability of results and the standardization of the surgical procedures. This study introduces a natural osteopromotive biomaterial that could enhance the quality of regenerated bone in a simple and efficient manner.

## Conclusion

Our findings showed that Moringa oleifera leaf extract has a positive effect on the quality and quantity of bone formed. The significant increase in the percentage of surface area of bone formed in test group compared to the size of the defect paves the way for further clinical research that could revolutionize the field of bone regeneration through a cost-effective, easily available osteopromotive material.

Moringa oleifera has been used in animal and clinical studies and has shown no side effects [35]. Animal studies testing the efficiency of Moringa oleifera on bone regeneration are recommended using large animal models, and in combination with different bone grafting materials.

## Data Availability

The datasets used during the current study are available from the corresponding author upon reasonable request.
